# Properties of Pain Assessment Tools for Use in People Living With Stroke: Systematic Review

**DOI:** 10.3389/fneur.2020.00792

**Published:** 2020-08-11

**Authors:** Sophie Amelia Edwards, Antreas Ioannou, Gail Carin-Levy, Eileen Cowey, Marian Brady, Sarah Morton, Tonje A. Sande, Gillian Mead, Terence J. Quinn

**Affiliations:** ^1^Institute of Cardiovascular and Medical Sciences, University of Glasgow, Glasgow, United Kingdom; ^2^Internal Medicine Department, Nicosia General Hospital, Strovolos, Cyprus; ^3^School of Health Sciences, Queen Margaret University, Edinburgh, United Kingdom; ^4^School of Medicine, University of Glasgow, Glasgow, United Kingdom; ^5^NMAHP Research Unit, Glasgow Caledonian University, Glasgow, United Kingdom; ^6^Centre for Clinical Brain Sciences, University of Edinburgh, Edinburgh, United Kingdom; ^7^Centre for Medical Informatics, Usher Institute, University of Edinburgh, Edinburgh, United Kingdom

**Keywords:** stroke, stroke care, pain, assessment, evaluation, psychometric

## Abstract

**Background:** Pain is a common problem after stroke and is associated with poor outcomes. There is no consensus on the optimal method of pain assessment in stroke. A review of the properties of tools should allow an evidence based approach to assessment.

**Objectives:** We aimed to systematically review published data on pain assessment tools used in stroke, with particular focus on classical test properties of: validity, reliability, feasibility, responsiveness.

**Methods:** We searched multiple, cross-disciplinary databases for studies evaluating properties of pain assessment tools used in stroke. We assessed risk of bias using the Quality Assessment of Diagnostic Accuracy Studies tool. We used a modified harvest plot to visually represent psychometric properties across tests.

**Results:** The search yielded 12 relevant articles, describing 10 different tools (*n* = 1,106 participants). There was substantial heterogeneity and an overall high risk of bias. The most commonly assessed property was validity (eight studies) and responsiveness the least (one study). There were no studies with a neuropathic or headache focus. Included tools were either scales or questionnaires. The most commonly assessed tool was the Faces Pain Scale (FPS) (6 studies). The limited number of papers precluded meaningful meta-analysis at level of pain assessment tool or pain syndrome. Even where common data were available across papers, results were conflicting e.g., two papers described FPS as feasible and two described the scale as having feasibility issues.

**Conclusion:** Robust data on the properties of pain assessment tools for stroke are limited. Our review highlights specific areas where evidence is lacking and could guide further research to identify the best tool(s) for assessing post-stroke pain. Improving feasibility of assessment in stroke survivors should be a future research target.

**Systematic Review Registration Number:** PROSPERO CRD42019160679

Available online at: https://www.crd.york.ac.uk/prospero/display_record.php?ID=CRD42019160679.

## Introduction

Pain is a common problem after stroke (1). Estimates of the frequency of pain vary across studies, depending on the population assessed and whether the focus is incident or prevalent pain. Large cohorts of mild to moderate stroke survivors suggest pain incidence of around 10% (2), while in smaller cohorts figures range from 30% during the first months (3), to 48% at 1 year (4) and 43% at 10 years (5) after stroke.

Post-stroke pain is associated with disability and reduced quality of life (1). It is independently associated with fatigue (6), depression (7) and has been strongly linked with suicidality (8, 9). Pain after stroke can have a variety of etiologies and manifestations, including: shoulder pain, headache, neuropathic pain and exacerbation of pre-existing pain. Pain symptoms can present at any point during stroke recovery and may progress to chronic pain if not recognized and treated appropriately.

The first step in managing post-stroke pain is recognition and measurement. However, management of pain has not always been given the same priority as other aspects of stroke care such as instituting secondary prevention (10). Pain assessment is a complicated task made more challenging in the context of stroke. Since pain is a subjective experience, self-report scales and questionnaires are the most commonly employed pain assessment tools in clinical practice and pain may be part of a more general health related quality of life assessment (11). However, stroke impairments such as cognitive decline and communication issues may make it difficult for stroke survivors to communicate the presence and experience of pain using these tools (12, 13). Other impairments such as visual issues or loss of motor skills may further complicate the use of self-completion questionnaires or visual analog scales.

Accepting these caveats, there is a range of pain assessment tools available that could be used with stroke survivors. Some are generic, some are specific to a certain pain syndrome and some are developed exclusively for stroke. At present there is no consensus on the best approach to assessing post-stroke pain and no standardized tool is recommended for research or practice (14). In the absence of a gold standard pain assessment in stroke survivors and with the great variety of assessment tools available, clinicians may struggle to know the most appropriate approach for their patients. The choice of assessment tools should be guided by evidence, particularly, the psychometric properties of the pain assessment tools available. Classical test features such as validity and responsiveness have been described for certain pain tools, however, equally important are end-user evaluations such as acceptability and feasibility within the person's healthcare setting.

A summary of psychometric properties of pain assessment tools could help clinicians and researchers choose the most appropriate measure, highlighting strengths and limitations and also showing where new evidence is needed. Thus, we conducted a systematic review to compare methods of pain assessment following stroke with a particular focus on properties of validity, reliability, feasibility, and responsiveness.

## Methods

We performed a systematic review, following best practice (15) and where appropriate Preferred Reporting Items for Systematic Reviews and Meta-Analyses (PRISMA) reporting guidance (16). Two assessors (SE, TQ) performed all aspects of title selection, data extraction and analyses with disagreements resolved through discussion.

As our focus was test properties, we structured our review question using the format recommended for test accuracy evidence synthesis (17).

- Index test: Any measure of pain that gives an objective read out.
- Reference standard: Any measure that provides data on the classical test properties of interest namely validity, reliability, feasibility and responsiveness.
- Condition: Stroke of any kind and at any stage in stroke journey.
- Setting: Any healthcare setting.

### Search Strategy

We searched the following databases, chosen to represent the various disciplines that may assess post-stroke pain: Medline (Ovid), Embase (Ovid), CINAHL (EBSCO) and PsychInfo (EBSCO). All were searched from inception to 1st May 2020. Search concepts were “stroke” and “pain” and “assessment.” We used validated search filters for “stroke” and “pain,” taken from the relevant Cochrane review group (Supplementary Materials). We complemented our search by contacting members of an international stroke pain research group to ensure we had not missed relevant studies.

We screened titles, abstracts and then full text to inform decisions on inclusion. Forward and backward citation searching was conducted for relevant studies using Web of Science functionality. As a test of search validity, we pre-specified two papers (one original research and one review) that should be returned on our literature search (1, 18). As a further test we cross-checked our included papers with a systematic review of pain assessment in aphasia, recognizing that the topics were distinct but were likely to have considerable overlap (14).

### Selection Criteria

The population of interest was adult stroke survivors at any stage of recovery. We did not include traumatic brain injury. If a mixed population was included, stroke had to represent more than 75% of the group. The test of interest was any form of pain assessment, including scales, questionnaires, observations and other patient reported outcome measures. Outcomes of interest were psychometric properties of the tools as defined below. We included studies of any quantitative design, conducted in any healthcare setting, noting setting as part of our data extraction. We only included studies published in peer reviewed journals but applied no other restrictions.

### Data Collection Process and Data Items

We designed and piloted a bespoke data collection form. We used the research paper that informed our internal validation for piloting (18).

We collected data on the following:

Study details: publication date, country, study design (*i.e.*, cross-sectional, prospective, retrospective), psychometric properties assessed (validity, feasibility, intra/inter-reliability, responsivity), sample size.

Stroke details: stroke classification (for example ischaemic or haemorrhagic), time since stroke, setting (classified as: acute stroke unit, rehabilitation, outpatient, community, using descriptions in the original paper), inclusion/exclusion criteria in original study, noting if there were specific exclusions relating to language or cognition.

Pain assessment: type of pain (see below), method(s) of pain assessment (*i.e.*, pain scales, questionnaires, stroke specific or generic), pain assessor(s) (*i.e.*, researcher or clinical discipline). For articles comparing multiple methods of pain assessment, we included all tools and recorded the primary pain assessment tool.

### Categorization of Pain Syndromes

We categorized pain using the following pre-specified labels: neuropathic, nociceptive (noting the site *i.e.*, lower limb), headache or experimental (*i.e.*, investigator induced pain). We classified stroke shoulder pain as a distinct category as it can include both nociceptive and neuropathic elements. Our pain classification was based on the description in the original paper. Where the nature of the pain syndrome was not clear, two reviewers (SE, TQ) discussed and came to consensus. For some papers, lack of detail precluded applying any label with certainty, and these were categorized as “non-specified.”

### Psychometric Properties

We were interested in the following psychometric properties: validity, reliability, feasibility, responsiveness. These were defined as (19, 20):

- Validity: the extent to which an instrument measures what is intended, in this case, is the tool a measure of pain? The concept of “accuracy” would be included as a measure of validity.
- Reliability: the internal consistency of an instrument, and the degree to which it is free from error on repeated. We included measures of inter-observer, intra-observer and internal reliability.
- Feasibility: usability, and acceptability of an instrument from the perspective of assessors and those being assessed.
- Responsiveness: the ability of the instrument to distinguish clinically important changes over time.

On initial scoping it became clear that a traditional quantitative meta-analysis would not be possible, due to the substantial clinical heterogeneity across studies in terms of populations assessed, methods used, nature of pain assessments and psychometric properties described. To allow cross-study comparisons, we created summary measures of the study findings at the level of the psychometric property studied. Our categorization was based on the conclusions of the original paper and was agreed by consensus of two assessors (SE, TQ). We classified results as positive, neutral or inconclusive.

### Risk of Bias

We assessed risk of bias for included studies at the outcome level. Two (SE, TQ) investigators individually assessed papers and agreed final grading. No single quality assessment tool would be suitable for the variety of methodologies that were included in our eligible papers. We elected to use the Quality Assessment of Diagnostic Accuracy Studies 2 (QUADAS-2) tool (21). QUADAS-2 is designed for assessing studies of test accuracy and uses a framework suited to our review with assessment of bias and applicability across four domains: patient selection, index tests, reference standard, flow and timing (17). As recommended, we took the original QUADAS-2 anchoring statements and modified to suit our review (modified domain questions included in Supplementary Materials). We used robvis R package software to create summary “traffic light” plots (22). Due to the limited number of studies and heterogeneity in summary measures we did not perform quantitative assessment for publication bias.

### Evidence Synthesis

We created two summary tables (Tables 1, 2): the first describes key characteristics of the included articles and the second summarizes their quantitative results. Our data were heterogeneous and required representation of differing constructs across various axes. To allow a visual representation that included pain syndrome, pain assessment tool and results of psychometric testing across various constructs we developed a visual plot using a modified harvest plot (23). We first created a matrix that plotted results by pain assessment tool (we created space in the plot for subcategorising by pain scales and questionnaires) against each psychometric property of interest. We color-coded according to pain type with one unit of plot space per study/experiment and then assigned the results of the study as positive (above a horizontal line of no effect), neutral (below the line) or inconclusive (crossing the line).

**Table 1:** Key Characteristics of included papers.

**Author/s**	**Study design**	**Psychometric properties assessed**	**Number included**	**Age (years) (mean, SD)**	**Stroke setting**	**Exclusion criteria**	**Type of pain**	**Pain assessment tool**	**Pain assessor**
1. Benaim (9)	Cross-sectional	Validity, reliability	127	63 ± 8	Rehabilitation	cognitive impairments, psychiatric disorders	Shoulder pain	FPS	Unknown
2. Chuang (24)	Prospective	Reliability	50	52.6 ± 11.0	Outpatient	other acute pain conditions, major medical problems, psychological impairments, aphasia	Arm/shoulder pain	v-NPRS-FPS	Clinical staff (rehabilitation physicians)
3. Dogan (25)	Case control	Validity	60 including non-stroke control (*n* = 30)	64.2 ± 9.42	Rehabilitation	Pre-existing pain conditions, cognitive impairment, aphasia	Shoulder pain	FPS	Unknown
4. Korner-Bitensky (26)	Cross-sectional	Validity	90	Not available	Rehabilitation	cognitive impairments, central post-stroke pain syndrome	Experimental (thermal)	10-cm v-VAS	Clinical staff (SLT), researcher
5. Price (18)	Case control	Feasibility, validity	144 including non-stroke controls (*n* = 48)	72.5 mean	Acute stroke unit	reduced conscious level or dysphasic	Experimental (pressure)	v/m/h-VAS	Researcher
6. Smith (27)	Retrospective	Feasibility	388	77 (IQR:66–86)	Acute stroke unit	subsequent strokes	Not specified	FPS and/or NRS	Clinical staff (Nurses)
7. Roosink (28)	Cross-sectional	Validity	19	57.5 ± 7. 5	Rehabilitation	other chronic pain conditions, neurological deficits	Shoulder pain	DN4	Unknown
8. Turner-Stokes (29)	Cross-sectional	Validity, reliability, feasibility	49	52.6 ± 3.1	Rehabilitation	not specified	Shoulder pain	AbilityQ, ShoulderQ	Researcher
9. Turner-Stokes (30)	Retrospective	Responsiveness	30	47.2 ± 2.2	Rehabilitation	not specified	Shoulder pain	AbilityQ, ShoulderQ	Clinical staff (Nurses)
10. Mandysová (31)	Cross-sectional	Validity, reliability, feasibility	80	71.0 ± 13.7 (range 22–94)	Acute stroke unit	reduced conscious level	Not specified	VAS/NRS, NRS, FPS-R	Researcher
11. Pomeroy (32)	Prospective	Reliability	33	74 (range 57–89)	Community	reduced conscious level, other pain conditions, no irregular pain medication, no neurological/MSK disorders	Shoulder pain	10-cm v-VAS	Clinical staff (physiotherapist)
12. Soares (33)	Cross-sectional	Reliability, validity	36	61 median (range 46–71.75)	Acute stroke unit	neurological disorders	Experimental (mechanical)	PACSLAC-II	Clinical staff (Neurology nurses)

*Study design and setting were categorized and agreed by two raters (SE, TQ)*.

*FPS, Faces Pain Scale; NRS/NPRS, Numerical Rating Scale; VAS, Visual Analog Scale, v-/m-/h-, vertical/mechanical/horizontal*.

*NPRS-FPS and VAS/NRS indicate combined versions of scales DN4, neuropathic pain diagnostic questionnaire; PACSLAC-II, Pain Assessment Scale for Seniors with Severe Dementia-II*.

*SLT, Speech and Language Therapy*.

*N.B. more comprehensive version of table is available in Supplementary Materials*.

**Table 2:** Summary of results from included articles.

**Author/s**	**Pain assessment (comparator)**	**Results**
1. Benaim (9)	FPS (VAS, VRS)	• *Validity*: Correlation of FPS with VAS and VRS in both left and right hemisphere stroke (*r* = 0.65–0.82) • *Reliability*: • *Inter-rater:K*:0.64 (SE = 0.11) and *K:*0.44 (0.09) in left and right hemisphere stroke respectively. • *Intra-rater:K*:0.74 (0.13) and *K*:0.53 (0.10) in left and right hemisphere stroke respectively. • *Feasibility*: FPS was preferred in left hemisphere stroke, VAS was preferred in right hemisphere stroke.
2. Chuang (24)	v-NPRS-FPS	• *Reliability (intra-rater):*ICC=0.82 (SE=0.81), [smallest real difference = 1.87]. • No significant systematic bias between repeated measurements for NPRS-FPS. • High level of stability and minimal temporal variation, range of limits of agreement (−2.50 to 1.90)
3. Dogan (25)	FPS (VAS, LPS, NRS)	• *Validity*: Correlation of FPS with other pain scales in both groups (*r* = 0.95–0.97 and 0.67–0.93, respectively).
4. Korner-Bitensky (26)	10-cm v-VAS	• *Validity*: No between group difference in pain discrimination (*p =* 0.75). • Repeated-measures ANOVA revealed no effect of group.
5. Price (18)	v/m/h-VAS FPRS, NRS	• *Feasibility*: Range (proportion) of stroke survivors able to complete various versions of VAS 65–47% (*P* < 0.001 in comparison to non-stroke controls) • Range (proportion) of more severe stroke (TACS) able to complete various versions of VAS 28–14% (*P* < 0.001 in comparison to other strokes)
6. Smith (27)	FPS, NRS	• *Feasibility*: 13.4% individuals unable to provide a meaningful response to either FPS or NRS. • *Validity*: Maximum NRS values correlated with length of stay (*r* = 0.29, *P* < 0.0001), stroke severity (*r* = 0.212, *P* = 0.0008), and number of sites of pain (*r* = 0.20, *P* = 0.007).
7. Roosink (28)	DN4 (NRS)	• *Validity*: DN4+ classified patients reported: constant pain [DN4+:*n* = 4 (44%); DN4-:*n* = 0] higher pain intensity [DN4+ = 4.7 (SD = 2.9); DN4- = 2.5 (SD = 2.4)] higher impact of pain on daily living DN4+ = 5.9 (SD = 4.8), DN4- = 2.0 (SD = 2.6) more frequent loss of cold sensation [DN4+: *n* = 7 (78%); DN4-: *n* = 2 (20%)] • Signs and symptoms suggestive of neuropathic or nociceptive pain corresponded to DN4+ and DN4- respectively.
8. Turner-Stokes (29)	AbilityQ, ShoulderQ (VAS)	• *Validity:* VAS agreement ± 1 on a 10-point scale was 36–59% with intraclass correlation coefficients 0.50–0.60 (*p* < 0.01). • *Reliability:* Agreement for individual questions 55–88%; *K:*0.07–0.79 • Repeatability of ShoulderQ 36–72%, *K:* 0.16–0.56. • *Feasibility: N* = 31 (63%) required help in completing AbilityQ.
9. Turner-Stokes (30)	AbilityQ, ShoulderQ (VGRS)	• **Responsiveness**: Changes on VGRS associated with verbal reports of improvement (*r*: 0.67, *P* < 0.001). • Responders demonstrated significant change in VGRS and verbal scores, whereas non-responder group did not. • A change in summed VGRS score of ≥3 showed 77% sensitivity and 91.3% specificity for identifying responders, with a positive predictive value of 93.3%. Summed VGRS scores of ≤2 had a negative predictive value of 73.3%.
10. Mandysová (31)	VAS/NRS, NRS, FPS-R	• *Validity: n* = 19 (24%) reported pain using at least one scale. • Spearman correlation was 0.997 (*p* < 0.001) between VAS/NRS and NRS. • *Feasibility:* NRS had the highest preference ranking (ranking first or second in 75% cases).
11. Pomeroy (32)	10-cm v-VAS	• *Inter-rater reliability:*ICC:0.79 for intensity, 0.75 for frequency and 0.62 for affective response. • Wide limits of agreement and significant rater bias reported for 6/27 ratings. • *Intra-rater reliability*:ICC:0.70 for intensity, 0.77 for frequency and 0.69 for affective response.
12. Soares (33)	PACSLAC-II	• *Validity*: PACSLAC-II differentiated 4.5-lb stimulus versus 2-lb (*p* = 0.03) or 0lb (*p* = 0.05). • *Reliability (internal)*: Cronbach α:0.87, 0.94, and 0.96 for weights of 0, 2, and 4.5 lb, respectively.

*FPS, Faces Pain Scale; NRS/NPRS, Numerical Rating Scale; VAS, Visual Analog Scale; LPS, Likert Pain Scale; FPRS, Four-point rating scale; v-/m-/h-, vertical/mechanical/horizontal, visual graphic rating scale (VGRS); NPRS-FPS and VAS/NRS indicate combined versions of scales DN4=neuropathic pain diagnostic questionnaire (DN4+, neuropathic pain reported; DN4-, no neuropathic pain reported); PACSLAC-II, Pain Assessment Scale for Seniors with Severe Dementia-II*.

## Results

**Figure 1:**
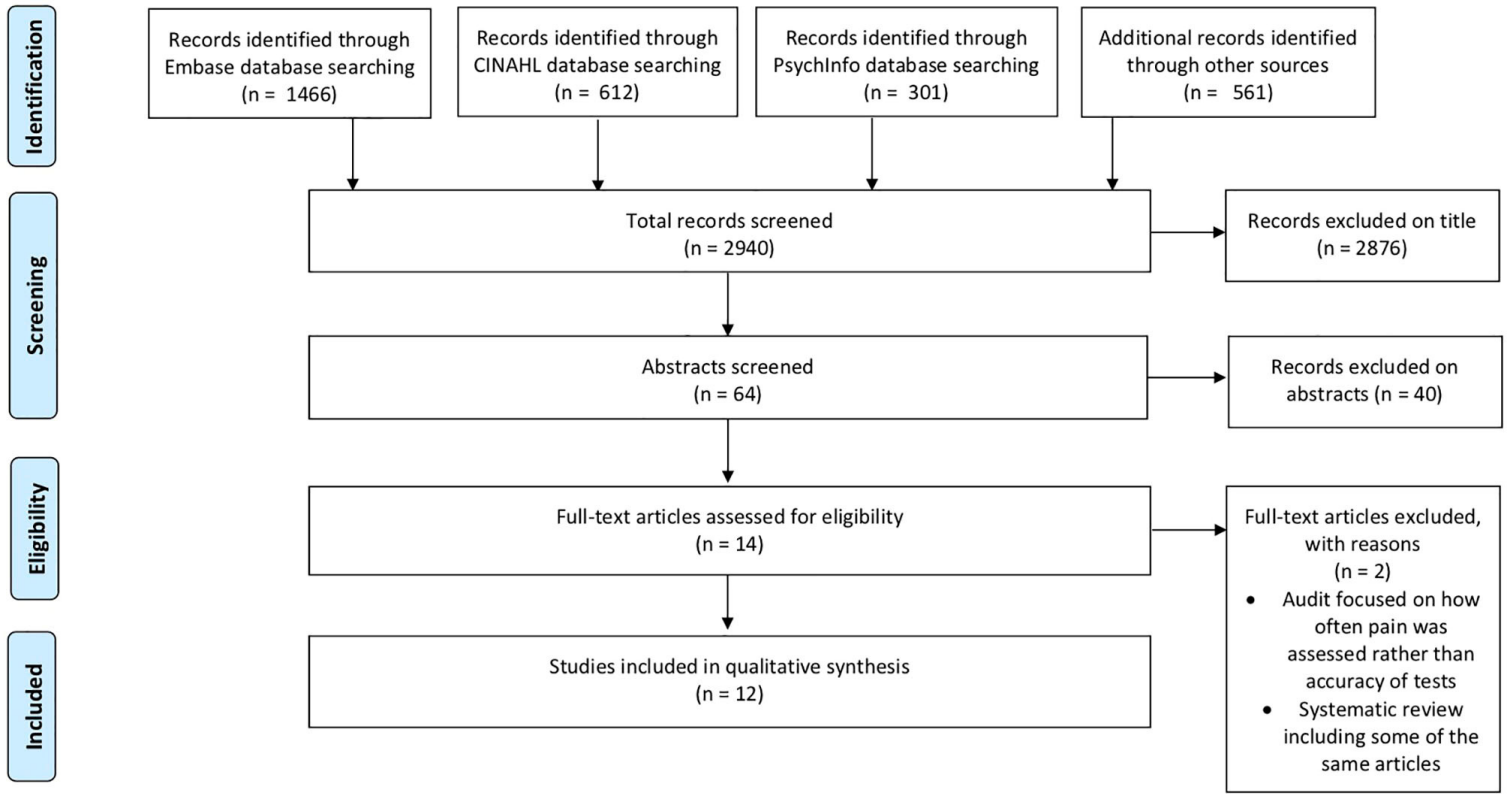
PRISMA Flow chart for selection of studies for systematic review. The first search was performed on 31st July 2019; to ensure the review was up to date we ran a repeat search on 08/05/2020. The PRISMA contains an aggregate of both searches.

The primary search yielded 2,851 articles, with 12 (9, 18, 24–33) papers (*n* = 1,106 participants) meeting the inclusion criteria (Figure 1). Our search results suggested a valid search as they included the two pre-selected papers and had all the relevant studies from the previous aphasia review. The number of participants in eligible papers ranged from 19 to 388. The most commonly employed design was cross-sectional (*n* = 6) with the majority of studies (*n* = 6) conducted in a rehabilitation setting (Table 1, Supplementary Materials).

In total, 10 different pain scales and questionnaires were assessed across the 12 studies (Table 1). These were: Visual Analog Scale (VAS [differing scales described as VAS]), the Faces Pain Scale (including a revised version), Numerical Rating Scale, and various combinations of these; the Pain Assessment Scale for Seniors with Severe Dementia-II (PACSLAC-II), and three questionnaires: AbilityQ, ShoulderQ and the neuropathic pain diagnostic questionnaire (DN4). Of the included assessments, only the ShoulderQ was developed specifically for stroke. The Faces Pain Scale was the most commonly reported, with a version of this scale used in six of the 12 studies.

Where a pain category was described, the most commonly studied was shoulder pain. Neuropathic pain and Headache were not studied, except possibly in those papers that did not differentiate pain type. There was heterogeneity in the tools assessed for each pain category, with no pain category having more than two studies using a common tool (Table 3).

**Table 3:** Cross-tabulation of pain assessment tool and post stroke pain syndrome.

	**Pain assessment tool**									
		**VAS**	**VAS-NRS**	**FPS**	**FPS-NRS**	**NRS**	**VRS**	**ShoulderQ**	**PACSLAC-11**	**DN4**
Post-stroke pain syndrome	Shoulder/arm pain	1	0	2	1	0	0	2	0	1
	Experimental	2	0	0	0	0	0	0	1	0
	Not specified	0	1	2	0	2	0	0	0	0
	Neuropathic	0	0	0	0	0	0	0	0	0
	Headache	0	0	0	0	0	0	0	0	0

*Each value represents use of a pain assessment tool by according to a post-stroke pain syndrome*.

*FPS, Faces Pain Scale; NRS/NPRS, Numerical Rating Scale; VAS, Visual Analog Scale; LPS, Likert Pain Scale; FPRS, Four-point rating scale; v-/m-/h-, vertical/mechanical/horizontal, visual graphic rating scale (VGRS), NPRS-FPS and VAS/NRS indicate combined versions of scales DN4, neuropathic pain diagnostic questionnaire (DN4+, neuropathic pain reported; DN4-, no neuropathic pain reported); PACSLAC-II, Pain Assessment Scale for Seniors with Severe Dementia-II*.

**Figure 2:**
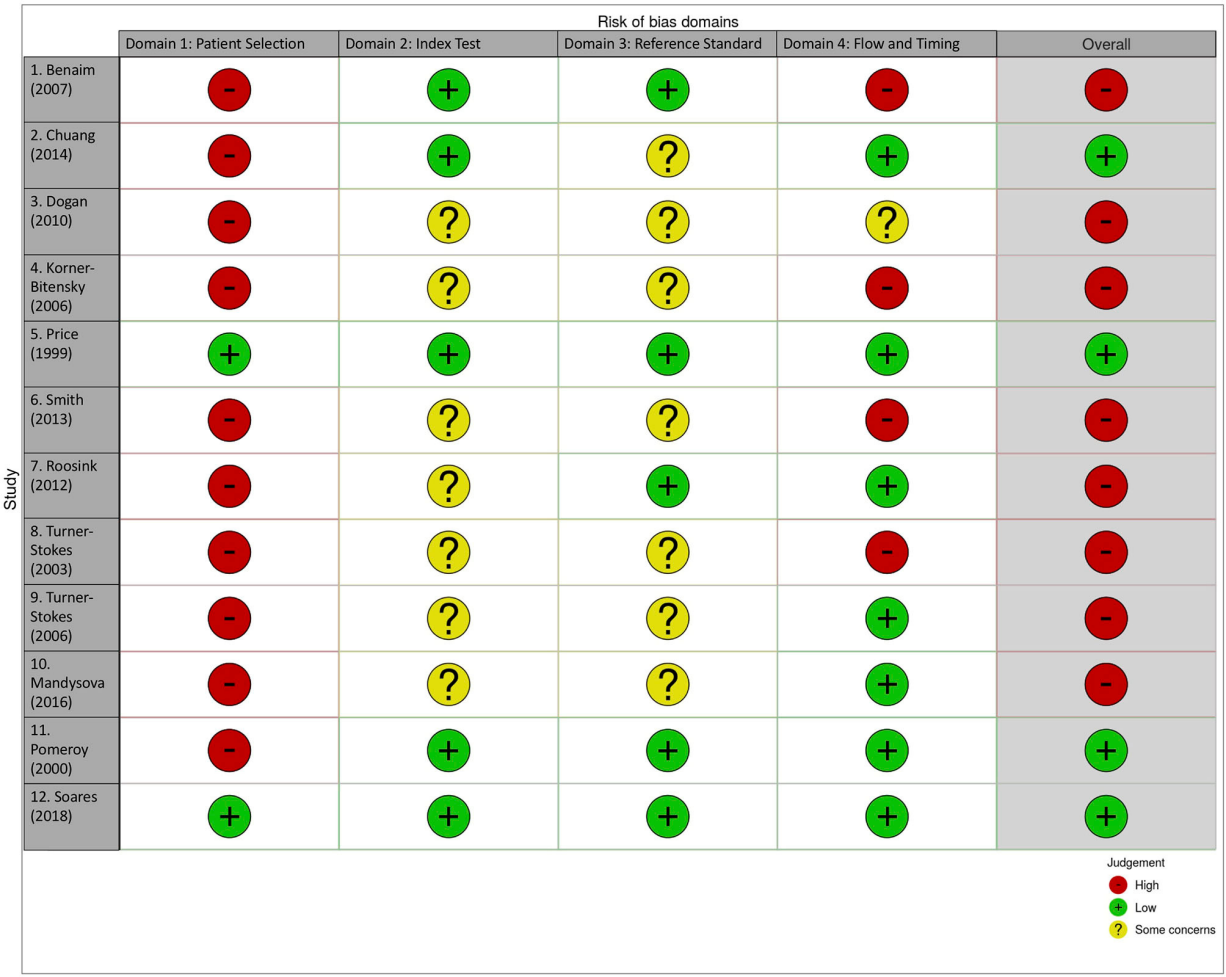
Traffic Light plot for risk of bias in individual studies.

There was a high risk of bias detected in the majority of included papers (*n* = 8; Figure 2). Highest risk of bias and issues with generalisability was seen for the domain of patient selection (*n* = 10; judged high risk). This was due to exclusion of patients for whom pain assessment would be expected in clinical practice, including those with pre-stroke pain (*n* = 5 papers), aphasia (*n* = 3) and cognitive impairment (*n* = 3). There was poor reporting of study methods relevant to the risk of bias assessment, particularly around blinding of results when a study compared scales. Only four papers were judged to have overall low risk of bias (18, 24, 32, 33).

**Figure 3:**
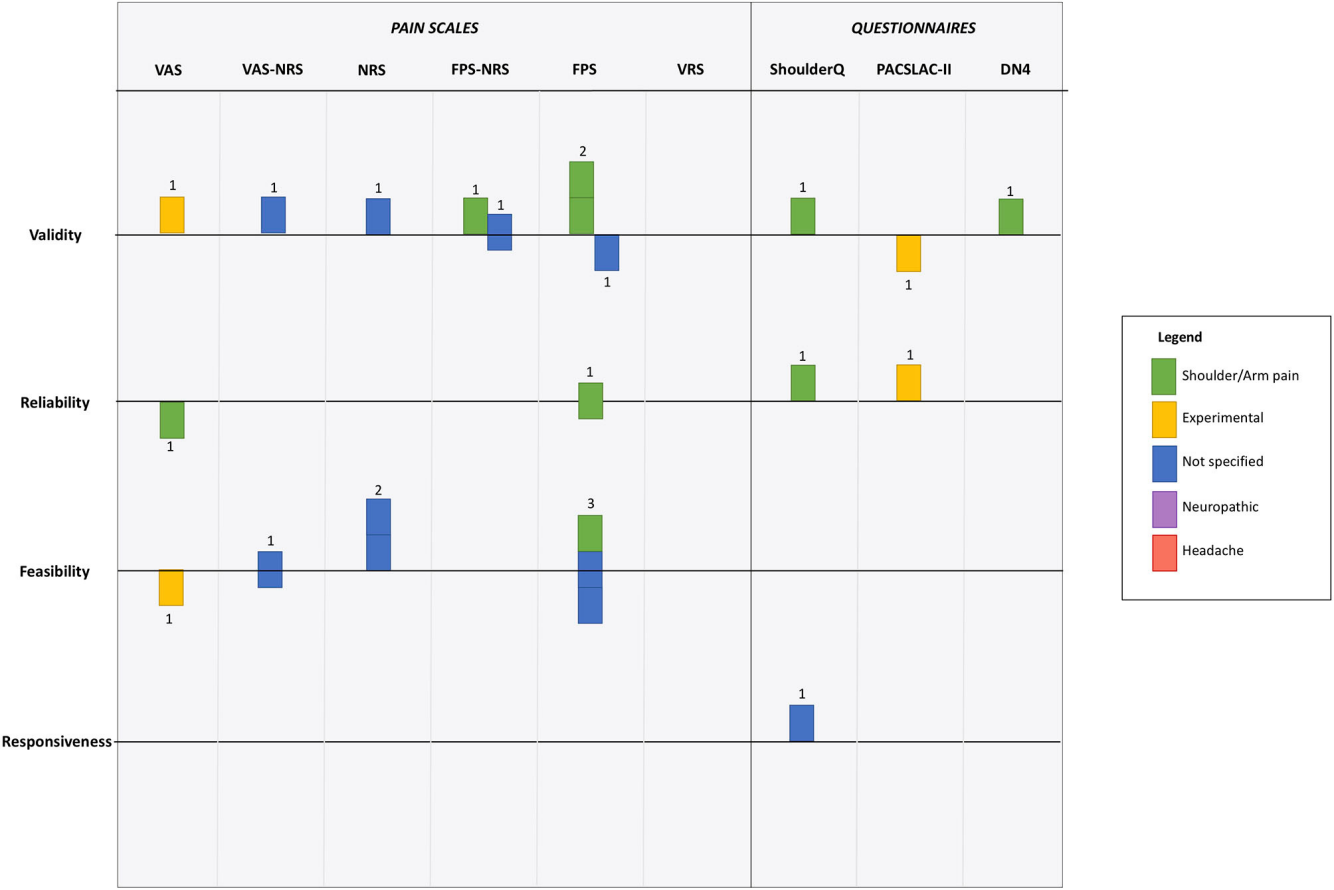
Harvest plot of psychometric evaluation of pain scale according to the 12 included studies. *Each unit represents a differing study. Color coding is used to represent differing pain types. Position around horizontal line describes paper conclusions regarding the property of interest, where above the line indicates “good,” below the line indicates “poor” and on the line indicates “uncertain.” Full description given in main manuscript. VAS, Visual Analog Scale; NRS, Numerical Rating Scale; FPS, Faces Pain Scale; VRS, Visual Rating Scale; ShoulderQ, Shoulder pain questionnaire; PACSLAC-II, Pain Assessment Scale for Seniors with Severe Dementia-II; DN4, neuropathic pain diagnostic* questionnaire.

We created a visual synthesis of the psychometric properties of the tools used to assess pain as a modified harvest plot (Figure 3). The harvest plot approach allows visual display of data across several axes in one figure. We represented each study as a single unit (square), and color coded based on pain type. A horizontal line that bisected each row was a line of uncertain effect, if a study claimed that the psychometric property of interest was “good” i.e., acceptable for clinical use then the study was placed above the line, if the paper reported that the study was “poor” i.e., would not be suitable it was placed below the line.

All psychometric domains of interest were reviewed by at least one study, although the statistical approach to these assessments varied. Validity was the psychometric property evaluated most frequently (*n* = 8), and responsiveness was only considered by one study. In general the pain scales assessed were judged to be valid measures by the authors of the studies, with only two studies reporting concerns around validity (Figure 3). A version of the Faces Pain Scale was the most commonly assessed, with evaluations of validity (*n* = 3), reliability (*n* = 3), and feasibility (*n* = 2). However, results were conflicting, for example feasibility of FPS was assessed as good, neutral and poor across the studies (Figure 3).

## Discussion

We aimed to systematically review the psychometrics of pain assessment tools when used with stroke survivors. We found a limited literature with substantial heterogeneity in the tools used, the research methods employed and the properties assessed. The available data were limited by risk of bias and modest sample sizes. Thus, we are unable to recommend a preferred tool based on published psychometric properties. However, through our evidence synthesis, we have highlighted important evidence gaps that can inform the direction of future research activity in the pain assessment space.

Our mapping of the evidence using the harvest plot demonstrates the many limitations in the evidence base. Of the four key psychometric properties, there was little information on reliability, and responsiveness. Even where there was a portfolio of papers on a single tool it was difficult to draw conclusions. There were more studies on visual scales than questionnaires, with few studies using a scale specifically developed for stroke and no studies with a neuropathic or headache pain focus.

Our findings of inconsistent and inconclusive evidence are not unique to stroke. A previous review of pain assessment in aphasia concluded that “a feasible, reliable and valid pain assessment instrument is not yet available” (14). Dementia is another clinical condition where pain is common but potentially difficult to assess. Although there is more published literature on dementia pain assessment tools (34), conclusions of reviews are similar “limited evidence about reliability, validity and clinical utility” (35). This seems a missed opportunity, as well as the clinical importance of looking for pain, quantitative pain assessment could be a useful research outcome (36).

Our assessment of risk of bias suggests common areas of concern particularly around reporting and generalisability. Exclusion of stroke survivors with aphasia, dementia or comorbidity threatens the external validity of study results. Similar exclusions have been demonstrated in other aspects of stroke assessment (37). Certain scales may not be suitable for all stroke impairments, but simply excluding those people who may struggle to complete an assessment creates bias in any resulting estimates (38).

Our review has several strengths. We performed a comprehensive search, followed best practice guidance and embedded internal validation steps. Given the disparate nature of relevant studies, we used non-traditional methods for evidence synthesis and assessment of quality. There are limitations to our approach. Despite internal and external validity steps we may have missed relevant papers. We were not able to perform quantitative meta-analysis either at an aggregate level or at the level of differing pain types, but instead used a relatively novel method of visual data synthesis. Our modified harvest plot approach gives a summary of the totality of the data across various axes, allowing for visual comparisons across tools. This approach could be applied in other complex reviews with substantial heterogeneity in the supporting literature.

Despite the prevalence of post-stroke pain, studies describing the best way to assess for this problem are limited in number and quality. Our evidence mapping and quality assessments highlight particular pain syndromes and tests that have no empirical evidence base. No pain assessment had sufficient data to be considered definitive and further, robust research for any pain tool would be a welcome addition.

In light of this uncertainty what conclusions can be made? Patient based scales, such as faces pain scale, seem to have the most supporting evidence and are a valid means to assess pain. Our review suggests there are many evidence gaps requiring future research, but methods to improve feasibility of assessment seem an important target.

## Data Availability Statement

All datasets presented in this study are included in the article/Supplementary Material.

## Author Contributions

SE contributed to all aspects of searching, data extraction and analysis, provided critical review, and contributed to draft manuscripts. AI assisted with data extraction, provided critical review, and contributed to draft manuscripts. GC-L provided critical review, assisted with formatting, and contributed to draft manuscripts. EC provided critical review, assisted with formatting, and contributed to draft manuscripts. MB provided critical review, expert aphasia advice, and contributed to draft manuscripts. SM provided critical review and contributed to draft manuscripts. TS provided critical review and contributed to draft manuscripts. GM devised the study question, coordinated the team, and contributed to draft manuscripts. TQ provided critical review and contributed to draft manuscripts. All authors contributed to the article and approved the submitted version.

## Conflict of Interest

The authors declare that the research was conducted in the absence of any commercial or financial relationships that could be construed as a potential conflict of interest.
