# Supplementary material for: Properties of Pain Assessment Tools for Use in People Living With Stroke: Systematic Review
Source: Front Neurol. 2020 Aug 11;11:792. doi: 10.3389/fneur.2020.00792 (PMC7431893; doi:10.3389/fneur.2020.00792)
Supplement: Supplementary file 1 [file Table_1.DOCX]

***Search Criteria***

**1) Embase**

| 1. | cerebrovascular disorders/ or exp basal ganglia cerebrovascular disease/ or exp brain ischemia/ or exp carotid artery diseases/ or exp cerebrovascular trauma/ or exp intracranial arterial diseases/ or exp intracranial arteriovenous malformations/ or exp "intracranial embolism and thrombosis"/ or exp intracranial hemorrhages/ or stroke/ or exp brain infarction/ or vertebral artery dissection/ |
| --- | --- |
| 2. | (stroke or poststroke or post stroke or cerebrovasc$ or brain vasc$ or cerebral vasc$ or cva$ or apoplex$ or SAH).tw. |
| 3. | ((brain$ or cerebr$ or cerebell$ or intracran$ or intracerebral) adj5 (isch?emi$ or infarct$ or thrombo$ or emboli$ or occlus$)).tw. |
| 4. | ((brain$ or cerebr$ or cerebell$ or intracerebral or intracranial or subarachnoid) adj5 (haemorrhage$ or hemorrhage$ or haematoma$ or hematoma$ or bleed$)).tw. |
| 5. | 1 or 2 or 3 or 4 |
| 6. | (Assess$ adj5 pain).mp. |
| 7. | (Measur$ adj5 pain).mp. |
| 8. | (Scale$ adj5 pain).mp. |
| 9. | (Rating adj5 pain).mp. |
| 10. | exp Pain Measurement/ |
| 11. | exp Pain/di |
| 12. | *Pain Measurement/mt |
| 13. | exp *Pain Measurement/ |
| 14. | (Pain adj3 tool$).mp. |
| 15. | 6 or 7 or 8 or 9 or 10 or 11 or 12 or 13 or 14 |
| 16. | 5 and 15 |

**2) CINAHL**

| stroke or cerebrovascular accident or cva or cerebral vascular event or cve  or transient ischaemic attack or tia (Title) |
| --- |
| *AND* |
| pain or pain assessment (Abstract) |

**3) PsychInfo**

| stroke or cerebrovascular accident or cva or cerebral vascular event or cve  or transient ischaemic attack or tia (Title) |
| --- |
| *AND* |
| pain or pain assessment (Abstract) |
