# Supplementary material for: Properties of Pain Assessment Tools for Use in People Living With Stroke: Systematic Review
Source: Front Neurol. 2020 Aug 11;11:792. doi: 10.3389/fneur.2020.00792 (PMC7431893; doi:10.3389/fneur.2020.00792)
Supplement: Supplementary file 3 [file Data_Sheet_1.PDF]

Search Criteria

1) Embase

1.	cerebrovascular disorders/ or exp basal ganglia cerebrovascular disease/ or exp brain ischemia/ or exp carotid artery diseases/ or exp cerebrovascular trauma/ or exp intracranial arterial diseases/ or exp intracranial arteriovenous malformations/ or exp "intracranial embolism and thrombosis"/ or exp intracranial hemorrhages/ or stroke/ or exp brain infarction/ or vertebral artery dissection/
2.	(stroke or poststroke or post stroke or cerebrovasc$ or brain vasc$ or cerebral vasc$ or cva$ or apoplex$ or SAH).tw.
3.	((brain$ or cerebr$ or cerebell$ or intracran$ or intracerebral) adj5 (isch?emi$ or infarct$ or thrombo$ or emboli$ or occlus$)).tw.
4.	((brain$ or cerebr$ or cerebell$ or intracerebral or intracranial or subarachnoid) adj5 (haemorrhage$ or hemorrhage$ or haematoma$ or hematoma$ or bleed$)).tw.
5.	1 or 2 or 3 or 4
6.	(Assess$ adj5 pain).mp.
7.	(Measur$ adj5 pain).mp.
8.	(Scale$ adj5 pain).mp.
9.	(Rating adj5 pain).mp.
10.	exp Pain Measurement/
11.	exp Pain/di
12.	*Pain Measurement/mt
13.	exp *Pain Measurement/
14.	(Pain adj3 tool$).mp.
15.	6 or 7 or 8 or 9 or 10 or 11 or 12 or 13 or 14
16.	5 and 15

2) CINAHL

stroke or cerebrovascular accident or cva or cerebral vascular event or cve or transient ischaemic attack or tia (Title)
AND
pain or pain assessment (Abstract)

3) PsychInfo

stroke or cerebrovascular accident or cva or cerebral vascular event or cve or transient ischaemic attack or tia (Title)
AND
pain or pain assessment (Abstract)
