# Supplementary material for: Properties of Pain Assessment Tools for Use in People Living With Stroke: Systematic Review
Source: Front Neurol. 2020 Aug 11;11:792. doi: 10.3389/fneur.2020.00792 (PMC7431893; doi:10.3389/fneur.2020.00792)
Supplement: Supplementary file 4 [file Data_Sheet_2.PDF]

	Title	Authors	Primary Aim	Database	Cited References	Times Cited	Year	Country	Study Design	Psychometric properties assessed	Number included	Age (years) (mean, SD)	Stroke Details				Pain Assessment		
													Type of stroke	Time since stroke	Stroke setting	Exclusion Criteria	Type of pain	Assessment tool	Assessor
**1. Benaim [9]**	Use of the Faces Pain Scale by left and right hemispheric stroke patients	Benaim *et al.*	assess the psychometric properties of a vertical FPS for evaluating shoulder pain in stroke patients	Embase	41	31	2007	France	cross-sectional study	validity, inter and intra-rater reliability	127	63 ± 8	first-ever stroke, unilateral MCA	any stage	rehabilitation setting	cognitive impairments, psychiatric disorders	Shoulder Pain	FPS	unknown
**2. Chuang [31]**	Relative and Absolute Reliability of a Vertical Numerical Pain Rating Scale Supplemented With a Faces Pain Scale After Stroke	Chuang *et al.*	investigate whether an NPRS supple- mented with an FPS (NPRS-FPS) would show good test-retest reliability in people with stroke	Embase	56	5	2014	Taiwan	prospective study	intra-rater reliability	50	52.6 ± 11.0	first-ever stroke, subacute and chronic stroke	> 3 months	outpatient setting	other acute pain conditions, major medical problems, psychological impairments, aphasia	Arm/Shoulder pain	v-NPRS-FPS	Clinical staff (Rehabilitation physicians)
**3. Dogan [26]**	The utility of the Faces Pain Scale in the assessment of shoulder pain in Turkish stroke patients: its relation with quality of life and psychologic status	Dogan *et al.*	investigate the utility of vertical FPS in the assessment of pain in stroke patients	Embase	40	8	2010	Turkey	case control	validity	60 including control (n=30)	64.2 ± 9.42	first-ever stroke	not specified	rehabilitation setting	Pre-existing pain conditions, cognitive impairment, aphasia	Shoulder Pain	FPS	unknown
**4. Korner-Bitensky [27]**	Eliciting Information on Differential Sensation of Heat in Those With and Without Poststroke Aphasia Using a Visual Analogue Scale	Korner-Bitensky *et al.*	determine whether those with poststroke aphasia could respond differentially to thermal stimuli of varying intensities using a standardized VAS	Embase	23	6	2006	Canada	cross-sectional study	validity	90	Not available	unilateral lesion, (some with aphasia)	not specified	rehabilitation setting	cognitive impairments, central post-stroke pain syndrome	Experimental (thermal)	10-cm v-VAS	Clinical staff (SLP), researcher
**5. Price [18]**	Can Stroke Patients Use Visual Analogue Scales?	Price *et al.*	investigate how stroke-related impairments could alter the ability of subjects to answer accurately	Embase	33	95	1999	UK	case control	feasibility, validity	144 including control (n= 48)	72.5 mean	not specified	< 6 months	acute stroke unit setting	reduced conscious level or dysphasic	Experiemental (pressure)	v/m/h-VAS	Researcher not involved in clinical care
**6. Smith [25]**	Inability to self-report pain after a stroke: A population-based study	Smith *et al.*	investigate the epidemiology of not being able to self-report pain after a stroke	Embase	17	8	2013	USA	retrospective	feasibility	388	77 (IQR:66–86)	Acute/Chronic	not specified	acute stroke unit setting	subsequent strokes	not specified	FPS and/or NRS	Clinical staff (nurses)
**7. Roosink [24]**	Classifying post-stroke shoulder pain: Can the DN4 be helpful?	Roosink *et al.*	explore whether the DN4 might be useful for the classification of PSSP subtypes	Embase	22	unknown	2012	Netherlands	cross-sectional study	validity	19	57.5 ±7. 5	unilateral brain infarction	> 6 months	rehabilitation setting	other chronic pain conditions, neurological deficits	Shoulder Pain	DN4	unknown
**8. Turner-Stokes (2003) [28]**	Screening for ability to complete a questionnaire: a preliminary evaluation of the AbilityQ and ShoulderQ for assessing shoulder pain in stroke patients	Turner-Stokes *et al.*	assess the repeatability of the AbilityQ – a screening tool to assess technical ability to complete a questionnaire – in patients with complex disabilities following stroke	Embase	12	16	2003	UK	cross-sectional study	validity, reliability, feasibility	49	52.6 ± 3.1	anterior or posterior circulation strokes	not specified	rehabilitation setting	not specified	Shoulder Pain	AbilityQ, ShoulderQ	Researcher not involved in clinical care
**9. Turner-Stokes (2006) [29]**	Assessment of shoulder pain in hemiplegia: Sensitivity of the ShoulderQ	Turner-Stokes *et al.*	assess the sensitivity of the ShoulderQ to clinical improvement in shoulder pain following multi-disciplinary intervention	Embase	7	14	2006	UK	retrospective	responsiveness	30	47.2 ± 2.2	presence of severe complex neurological disability (any cause)	not specified	rehabilitation setting	not specified	Shoulder Pain	AbilityQ, ShoulderQ	Clinical staff (nurses)
**10. Mandysova [30]**	A comparison of three self-report pain scales in Czech patients with stroke	Mandysová *et al.*	determine the performance of three self-report pain scales	CINALH	25	unknown	2016	Czech Republic	cross-sectional study	validity, reliability, feasibility	80	71.0 ± 13.7 (range 22–94)	not specified	not specified	acute stroke unit setting	reduced conscious level	not specified	VAS/NRS, NRS, FPS-R	Researcher not involved in clinical care
**11. Pomeroy [32]**	Reliability of a measure of post-stroke shoulder pain in patients with and without aphasia and/or unilateral spatial neglect	Pomeroy *et al.*	determine the inter/intra-rater reliability of expert physiotherapists (PTs) measuring post-stroke shoulder pain with 100 mm vertical visual analogue scales (VAS; intensity, frequency and affective response) and a categorical site-of-pain scale.	CINALH	33	3	2000	UK	prospective study	inter/intra-rater reliability	33	74 (range 57–89)	not specified	> 6 months	community setting	reduced conscious level, other pain conditions, no irregular pain medication, no neurological/MSK disorders	Shoulder Pain	10-cm v-VAS	Physioytherapist
**12. Soares [33]**	Experimental pain assessment in patients with poststroke aphasia	Soares *et al.*	evaluate an observational-behavioral pain tool among individuals with acute poststroke aphasia.	Forward/ Backward searching	24	1	2018	USA	cross-sectional study	reliability, validity	36	61 median (range 46 - 71.75)	presence of post-stroke aphaisa	< 7 days	acute stroke unit setting	neurological disorders	Experimental (mechanical)	PACSLAC-II	Clinical staff (Neurology nurses)
